# Hemichorea-hemiballismus as the initial manifestation of symptomatic middle cerebral artery dissection: A case report

**DOI:** 10.1097/MD.0000000000022116

**Published:** 2020-09-04

**Authors:** Hanfeng Chen, Ziqi Xu

**Affiliations:** Department of Neurology, The First Affiliated Hospital, College of Medicine, Zhejiang University, Hangzhou, China.

**Keywords:** dissection, hemichorea–hemiballismus, high-resolution magnetic resonance imaging, middle cerebral artery, movement disorder, stroke

## Abstract

**Introduction:**

Hemichorea-hemiballismus, which spans a spectrum of involuntary, continuous, nonpatterned movement involving one side of the body, can emerge as the initial manifestation of acute ischemic stroke. However, because of its rarity in the community, the diagnosis and treatment are often delayed.

**Patient concerns:**

We report a unique case of a 47-year-old female who presented with acute onset hemichorea-hemiballismus. No obvious focal sign apart from involuntary, continuous, nonpatterned movement of her left arm and leg was presented.

**Diagnosis:**

Initial diffusion-weighted magnetic resonance imaging (MRI) was negative but significant increase of blood flow velocity in the right middle cerebral artery (MCA) stem was revealed by transcranial doppler sonography. Repeated MRI showed acute infarction in the contralateral globus pallidus. Isolated dissection of the right MCA typified by intimal flap with double lumen was identified by digital subtraction angiography and high-resolution magnetic resonance imaging (HR-MRI).

**Interventions:**

The patient was initially treated with dual antiplatelet agents but the uncontrollable movement deteriorated during hospitalization. Antithrombotic therapy was then intensified with combination of tirofiban and low-molecular-weight heparin. Other symptomatic treatment included volume expansion with colloidal fluid to improve cerebral perfusion. Her involuntary movement gradually diminished and the patient was discharged with rivaroxaban 15 mg/daily.

**Outcomes:**

The patient had recovered with significant reduction in her hemichorea-hemiballismus. Three-month follow-up HR-MRI showed complete resolution of the MCA dissection lesions.

**Conclusion:**

Prompt recognition of acute onset hemichorea-hemiballismus as the manifestation of acute ischemic stroke in appropriate clinical setting may reduce diagnostic delay. Multiple imaging techniques including cerebral digital subtraction angiography and HR-MRI can be applied to diagnosis and further clarify the mechanism of stroke, which facilitate in selection of secondary prevention therapies.

## Abstract 2

Supplemental Digital Content is available in the text

## 1 Introduction

Post-stroke hyperkinetic movement disorder can occur immediately at the onset of acute stroke, or occasionally be delayed-onset and progressive, which resulting in diagnostic difficulty and error because of its clinical rarity. Hemichorea-hemiballismus, which spans a spectrum of involuntary, continuous, nonpatterned movement involving one side of the body, is the most frequent post-stroke hyperkinetic movement disorder and always emerges as acute manifestation. Previous reports suggest that the most common subtype of stroke leading to hemichorea-hemiballismus is cerebral small vessel disease with small deep infarcts in contralateral subthalamic nucleus or adjacent structures.^[1–3]^ But cardiac cerebral embolism, as well as large vessel atherothrombotic stroke with cortical or watershed zone infarcts, have also been reported.^[2,4,5]^ Isolated middle cerebral artery (MCA) dissection as an uncommon vasculopathy can lead to ischemic stroke by mechanism of thromboembolism, hypoperfusion, or rarely, local branch occlusion.^[6,7]^ Herein, we report a case of acute onset hemichorea-hemiballismus as the initial manifestation of symptomatic MCA dissection, which resolved after treatment with antithrombotic therapy.

## 2 Case presentation

A 47-year-old female was admitted to our hospital for evaluation of involuntary movements of her left side. She was woken up by the involuntary movements of her left limbs during sleep. The movement gradually increased in intensity and reached a plateau approximately one day before her clinic visit. There was no headache, language difficulty, visual disturbance, or paraesthesia. Her past medical history revealed two artificially induced abortions during the youth and a cesarean section 20 years ago. She was found to have hypertension 10 years ago and was prescribed with amlodipine 5 mg once daily. Her family history was negative for any neurological disorder and she did not have any history of trauma, diabetes, and smoking. On neurological examination, the movements involving her left arm and leg were involuntary, continuous and nonpatterned, which were consistent with hemichorea-hemiballismus (Video 1 and 2). Muscle strengths were all 5/5 and there were no cerebellar findings. The cranial nerves were intact and all modalities of sensation were normal.

**Figure 1:**
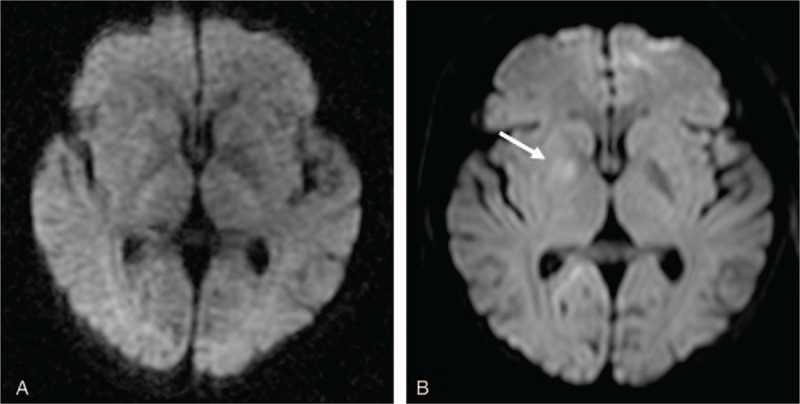
A, Initial diffusion-weighted MRI at presentation shows the absence of restricted diffusion. B, Diffusion-weighted MRI after deterioration of the symptom shows restricted diffusion in the right globus pallidus (arrow). MRI = magnetic resonance imaging.

**Figure 2:**
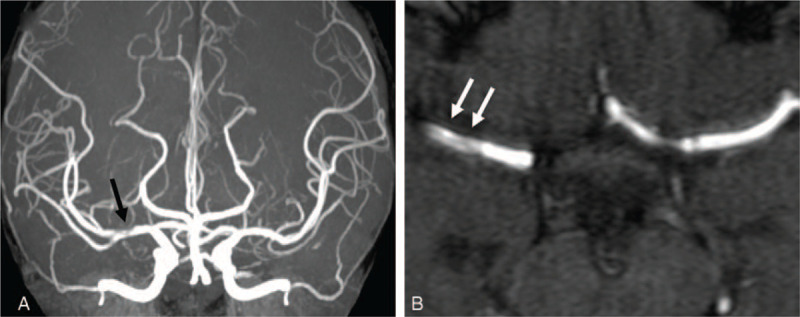
A, Time-of-flight MR angiography demonstrates narrowing of the horizontal segment of the right MCA (black arrow). B, Axial view of the source images shows the double lumen sign, although it is not obvious (arrows). MCA = middle cerebral artery, MR = magnetic resonance.

**Figure 3:**
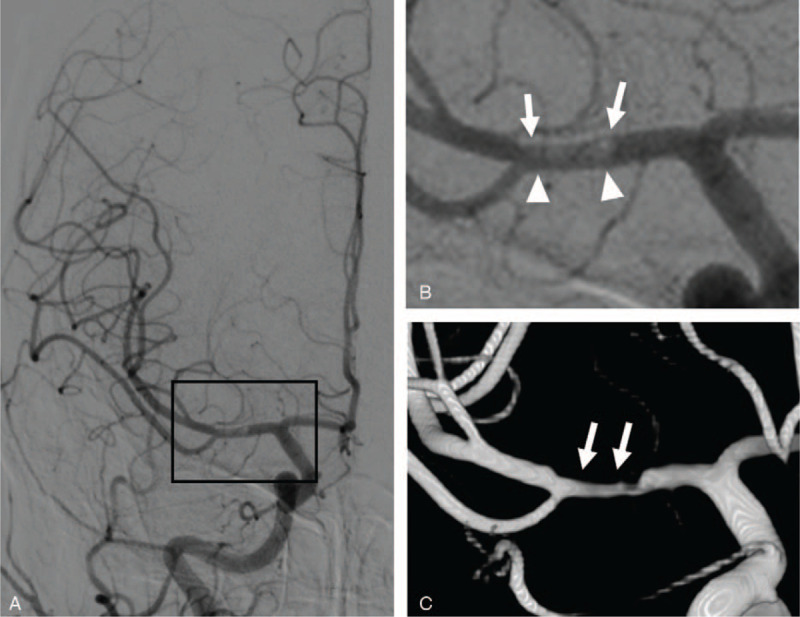
A-B, Pseudo-lumen with probable thrombus (arrows) adjacent to lenticulostriate arteries is presented in the cerebral DSA. Note the arrowheads point to the true lumen. C, Three-dimensional reconstruction image shows irregular luminal changes in the horizontal segment of MCA (arrows) consistent with arterial dissection. DSA = digital subtraction angiography, MCA = middle cerebral artery.

The patient underwent two conventional electroencephalograms that were normal. Emergency cranial computed tomography (CT) had excluded hemorrhage and magnetic resonance imaging (MRI) failed to reveal more diagnostic information (Fig. 1A). Extensive laboratory examinations including complete blood count, glucose, electrolytes, coagulation studies, assessment of hepatic, renal and thyroid functions were unremarkable (Table 1). Given the acute onset of the clinical manifestation, ischemic cerebrovascular disease was considered to be the most probable diagnosis despite negative diffusion-weighted imaging. Dual antiplatelet therapy combined with statin treatment were initiated and the patient was transferred to department of neurology for detailed evaluation. A significant increase of blood flow velocity in the right MCA stem was revealed by transcranial doppler sonography. Time-of-flight MR angiography on day 4 after admission showed segment narrowing of the right MCA (Fig. 2A) and the double lumen sign (Fig. 2B), on the basis of which isolated MCA dissection was strongly suggested. In order to evaluate the criminal intracranial artery lesion and assess any comorbidities that may contribute to the vasculopathy, further imaging examinations and laboratory tests were conducted. Immunologic blood tests showed slightly increased erythrocyte sedimentation rate and antiphospholipid antibody assessment revealed abnormal values, all of which dropped to normal level on the day of discharge (Tables 1 and 2). Lung CT, echocardiography and abdominal ultrasonography were all unremarkable. In comparison with Time-of-flight MR angiography, we performed the cerebral digital subtraction angiography on day 10 that showed a definite double lumen, diagnostic of arterial dissection, at the horizontal segment of the right MCA (Fig. 3). The pseudo-lumen was located dorsal to the true lumen and based on this anatomical feature, 1 or more of the lenticulostriate arteries might be blocked thus affecting blood perfusion in basal ganglia.

**Table 1:**
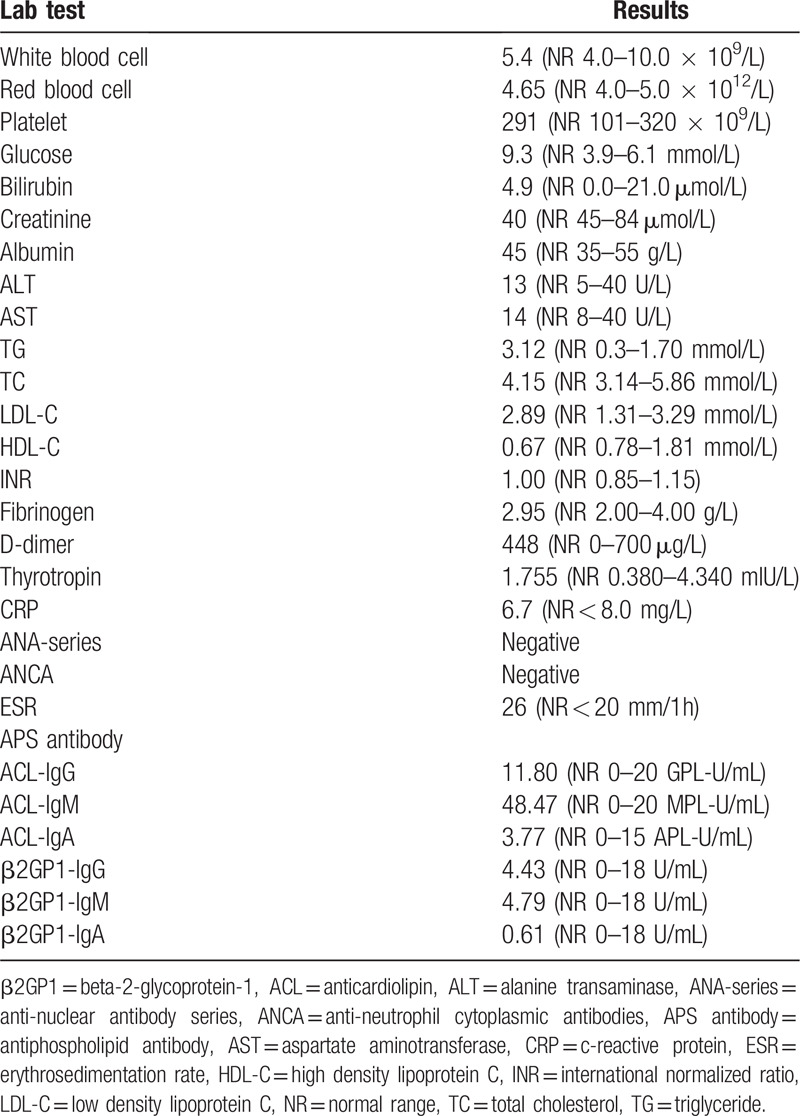
Initial comprehensive laboratory examinations.

**Table 2:**
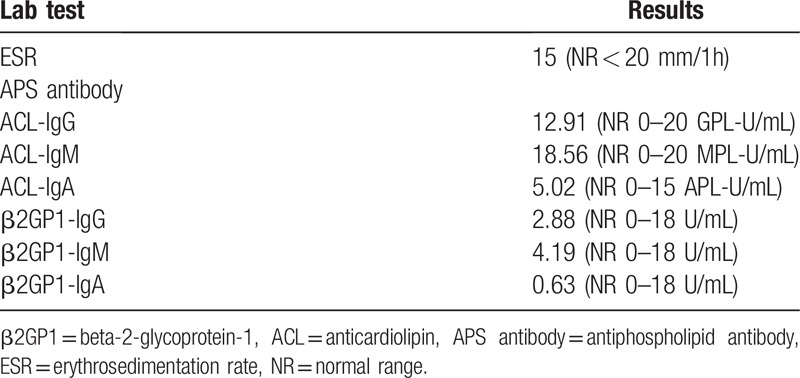
Repeated laboratory examinations on the day of discharge.

**Figure 4:**
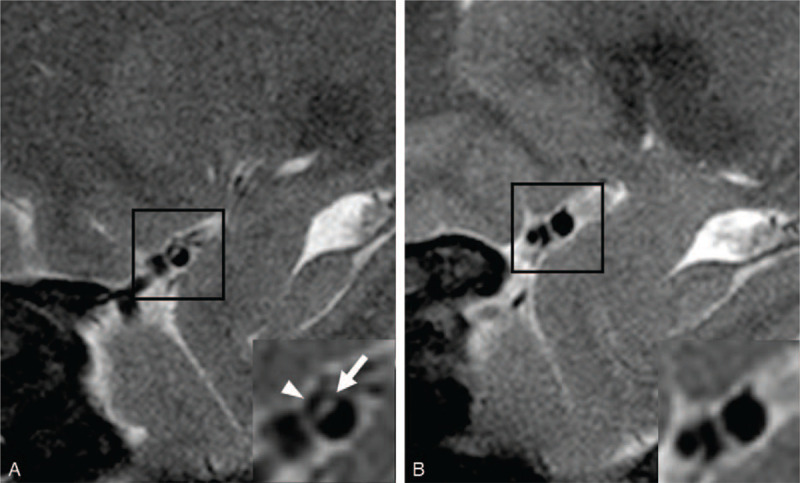
A, T2-weighted HR-MRI shows the intimal flap in the right MCA (arrow). Note the dorsally-located pseudo-lumen with absence of intramural hemorrhage (arrowhead). B, T2-weighted HR-MRI at three-month revisit shows resolution of the MCA dissection lesion. HR-MRI = high-resolution magnetic resonance imaging, MCA = middle cerebral artery.

On the 12th day of hospitalization, the patient's symptom deteriorated unpredictably with more prominent involuntary movements on her left sides. A repeated MRI demonstrated restricted diffusion within the right basal ganglia, specifically the globus pallidus (Fig. 1B). Intensified antithrombotic strategy with combination of tirofiban and low-molecular-weight heparin was adjusted to halt thrombus propagation in the pseudo-lumen. Volume expansion therapy with colloidal fluid was also prescribed to improve cerebral perfusion. The involuntary movements got diminished after treatment but remained stable during the follow-up period. High-resolution magnetic resonance imaging (HR-MRI) covering the MCA stem was performed on day 14 and revealed the intimal flap with tapered pseudo-lumen in the absence of intramural hemorrhage (Fig. 4A). By hospital day 16, the patient was discharged and treated with rivaroxaban 15 mg/daily. The involuntary movements gradually diminished over the next 3 months but persisted with slightly quality-of-life affected. Three-month follow-up vessel wall HR-MRI showed complete resolution of the MCA dissection lesions (Fig. 4B).

## 3 Discussion and conclusion

Hemichorea or hemiballismus is relatively rare movement disorder characterized by involuntary, brief, jerky, irregular and unpredictable contractions of muscle groups involving only 1 side of the body. Commonly, hemichorea and hemiballismus coexist in the same patient and are presumed to share the pathophysiology.^[8]^ Difference between them is mostly in movement phenomenology——involuntary movement that are proximal and large amplitude is defined as hemiballismus, while hemichorea is distally affected with relatively small amplitude. Thus it would seem more sensible to use the term ‘hemichorea-hemiballismus’ to describe these symptoms.

A variety of conditions can cause acquired hemichorea-hemiballismus syndrome.^[9–11]^ These include structural damage to deep brain structures (cerebrovascular disease,^[12]^ infection, trauma, neoplasia), or be associated with autoimmune/inflammatory disorders (antiphospholipid antibody syndrome,^[13]^ paraneoplastic chorea),^[14]^ metabolic derangement (nonketotic hyperglycemia),^[15,16]^ or exposure to certain drugs (dopamine agonist or phenytoin). As shared with the same pattern of movements regardless of its underlying etiology, the differential diagnosis of hemichorea-hemiballismus syndrome relies on accompanying features, age, mode of onset and laboratory or imaging examinations. In our patient, the initial diagnosis of ischemic stroke was based on experienced clinical evaluation while lacking of explicit neuroimaging. Fluctuation of the symptoms and diminishment of the involuntary movements after medical treatment further supported the diagnosis. Repeated MRI confirmed infarction of the globus pallidus. According to previous literature, the incidence of hemichorea-hemiballismus in acute ischemic stroke ranges between 0.4% and 0.54%,^[1,17]^ with a prevalence of 1%.^[1]^ In contrast to traditional textbook concept, a lesion of the subthalamic nucleus is found in only a minority of cases, other reported localizations include the caudate nucleus, putamen, thalamus, globus pallidus, subcortical white matter and cortex.^[2,10,17]^ It is believed that a complex network with reciprocal connections to several distinct brain regions involving the basal ganglia and subthalamic nucleus is responsible for high-level motor control. Disruption of this functional connectivity is postulated to produce characteristic movements of hemichorea-hemiballismus.^[17–19]^ To test this hypothesis, 29 separate cases of stroke-induced hemichorea-hemiballismus was included in the research and validated technique termed lesion network mapping was applied. The result demonstrated that the causative lesions, while anatomically heterogeneous, localize to a single network with shared functional connectivity to the posterolateral putamen.^[20]^ However, hemichorea-hemiballismus is still relatively rare even when the presumed network is markedly damaged. This observation might be explained by individual susceptibility and brain plasticity.^[21]^

Typically, the mechanism and classification of ischemic stroke can be inferred from clinical presentation and infarction distribution pattern. As the structural lesion responsible for hemichorea-hemiballismus is always small and deep-situated, the most common subtype of stroke is cerebral small vessel occlusion.^[1–3]^ But cardiac cerebral embolism, as well as large vessel atherothrombotic stroke with cortical or watershed zone infarcts, have also been reported.^[2,4,5]^ Although our patient had traditional vascular risk factors, cerebral digital subtraction angiography and vessel wall HR-MRI indicated that the lesion of MCA stem is non-atherothrombotic but in favor of arterial dissection. Resolution of the MCA lesion after 3 month antithrombotic therapy further confirmed the diagnosis. In clinical practice, isolated MCA dissection is an extremely rare clinical entity which poses a challenge to correct diagnosis for neurologists. Typical angiographic findings of MCA dissection are similar to dissection of the extracranial arteries, including double lumen by the presence of an intimal flap (string sign), irregular stenosis, pseudoaneurysm, and even total occlusion.^[22]^ Recently, HR-MRI has emerged as a useful technique, making it feasible to visualize the intimal flap and pseudo-lumen with intraluminal hemorrhage in vivo.^[23]^ To best of our knowledge, acute onset hemichorea-hemiballismus due to isolated MCA dissection has never been reported before. As separation of the arterial wall layers, the penetrating lenticulostriate arteries which mostly arose from the upper dorsal part of the MCA wall was occluded by local thrombus in the pseudolumen. Thus extension and regression of the thrombus over the orifice of lenticulostriate arteries directly influence distal perfusion in the basal ganglia, which interpret the fluctuation of symptom and effectiveness in medical therapy. As our patient denied any history of trauma and comprehensive diagnostic work was unremarkable with only transient anticardiolipin antibodies, which failed to fulfill the international sydney consensus diagnostic criteria for definite antiphospholipid syndrome,^[24]^ the etiology of MCA dissection remains uncertain.

The optimal treatment for patients with intracranial artery dissection is controversial. No randomized trials but only observational studies with small sample sizes are available, thus providing a very low level of evidence. So currently the treatment for intracranial artery dissection is based on the experience from extracranial artery dissection management, including thrombolysis, antithrombotic treatment, surgical repair, and endovascular interventions.^[7]^ The choice of antithrombotic treatment, whether anticoagulant or antiplatelet, in patients with intracranial artery dissection has been assessed only in some observational studies.^[25,26]^ The results were consistent with evidence from extracranial artery dissection management, suggesting no advantage of anticoagulation over antiplatelet treatment.^[27,28]^ Endovascular intervention or surgical repair has been recommended for patients who complicated with subarachnoid hemorrhage or have recurrent ischemia despite antithrombotic therapy.^[7,29]^ Our patient was treated with antiplatelet agents initially. We tentatively adjusted to combined use of antiplatelet and anticoagulation drugs for several days when symptoms deteriorated and then transitioned to anticoagulation therapy which lasted for 3 months. As irreversible ischemia was eventually occurred and demonstrated by the repeated MRI, slight choreiform movement persisted. Findings from literature review indicate that the prognosis of stroke-induced hemichorea-hemiballismus is related to lesion localization. Complete symptom resolution occurs in around 50% of cases with infarctions in the basal ganglia circuit, whereas subthalamic lesions result in persistent impairment.^[1,17]^ Dopamine blocker such as haloperidol and tetrabenazine have proven effective to lessen the involuntary movements.^[19,30–32]^ Although evidence is limited, symptom resolution with antiepileptic drugs such as clonazepam, sodium valproate, and topiramate were also reported in small, uncontrolled number of patients.^[33,34]^

In conclusion, we report a unique case of ischemic stroke presenting with acute onset hemichorea-hemiballismus, where basal ganglia perforating arteries were occluded due to isolate MCA dissection. Prompt recognition of this movement disorder in the appropriate clinical setting may reduce diagnostic delay and minimize functional impairment by early treatment.

## Author contributions

HFC designed and wrote the manuscript. HFC, ZQX examined the patient and analyzed the neuroimages. ZQX designed the case report and helped draft the manuscript. All authors read and approved the final manuscript.

**Conceptualization:** Ziqi Xu.

**Data curation:** Hanfeng Chen.

**Formal analysis:** Hanfeng Chen.

**Investigation:** Hanfeng Chen, Ziqi Xu.

**Methodology:** Hanfeng Chen.

**Resources:** Hanfeng Chen.

**Validation:** Hanfeng Chen, Ziqi Xu.

**Visualization:** Hanfeng Chen.

**Writing – original draft:** Hanfeng Chen.

**Writing – review & editing:** Ziqi Xu.

## Supplementary Material

Supplemental Digital Content

## Supplementary Material

Supplemental Digital Content
